# Metabolic requirements of CD160 expressing memory‐like NK cells in Gram‐negative bacterial infection

**DOI:** 10.1002/cti2.1513

**Published:** 2024-07-02

**Authors:** Anucha Preechanukul, Natnaree Saiprom, Kitilak Rochaikun, Boonthanom Moonmueangsan, Rungnapa Phunpang, Orawan Ottiwet, Yuphin Kongphrai, Soonthon Wapee, Rachan Janon, Susanna Dunachie, Barbara Kronsteiner, Narisara Chantratita

**Affiliations:** ^1^ Department of Microbiology and Immunology, Faculty of Tropical Medicine Mahidol University Bangkok Thailand; ^2^ Division of Infection and Immunity University College London London UK; ^3^ Department of Medical Technology and Clinical Pathology Mukdahan Hospital Mukdahan Thailand; ^4^ Department of Medicine Mukdahan Hospital Mukdahan Thailand; ^5^ Mahidol‐Oxford Tropical Medicine Research Unit Mahidol University Bangkok Thailand; ^6^ Peter Medawar Building for Pathogen Research, Nuffield Department of Clinical Medicine University of Oxford Oxford UK; ^7^ Nuffield Department of Clinical Medicine, NDM Centre for Global Health Research University of Oxford Oxford UK; ^8^ Oxford University Hospitals NHS Foundation Trust Oxford UK

**Keywords:** Gram‐negative bacterial infection, immunometabolism, melioidosis, NK cell, NK cell memory

## Abstract

**Objective:**

Unique metabolic requirements accompany the development and functional fates of immune cells. How cellular metabolism is important in natural killer (NK) cells and their memory‐like differentiation in bacterial infections remains elusive.

**Methods:**

Here, we utilise our established NK cell memory assay to investigate the metabolic requirement for memory‐like NK cell formation and function in response to the Gram‐negative intracellular bacteria *Burkholderia pseudomallei* (BP), the causative agent of melioidosis.

**Results:**

We demonstrate that CD160^+^ memory‐like NK cells upon BP stimulation upregulate glucose and amino acid transporters in a cohort of recovered melioidosis patients which is maintained at least 3‐month post‐hospital admission. Using an *in vitro* assay, human BP‐specific CD160^+^ memory‐like NK cells show metabolic priming including increased expression of glucose and amino acid transporters with elevated glucose uptake, increased mTOR activation and mitochondrial membrane potential upon BP re‐stimulation. Antigen‐specific and cytokine‐induced IFN‐γ production of this memory‐like NK cell subset are highly dependent on oxidative phosphorylation (OXPHOS) with some dependency on glycolysis, whereas the formation of CD160^+^ memory‐like NK cells *in vitro* is dependent on fatty acid oxidation and OXPHOS and further increased by metformin.

**Conclusion:**

This study reveals the link between metabolism and cellular function of memory‐like NK cells, which can be exploited for vaccine design and for monitoring protection against Gram‐negative bacterial infection.

## Graphical abstract

This study reveals the link between metabolism and cellular function of memory‐like NK cells in melioidosis. We demonstrate that CD160^+^ memory‐like NK cells upon *Burkholderia pseudomallei* (BP) stimulation upregulate glucose and amino acid transporters in a cohort of recovered melioidosis patients. Using an *in vitro* assay, human BP‐specific CD160^+^ memory‐like NK cells show metabolic priming including increased expression of glucose and amino acid transporters with elevated glucose uptake, increased mTOR activation and mitochondrial membrane potential upon BP re‐stimulation.

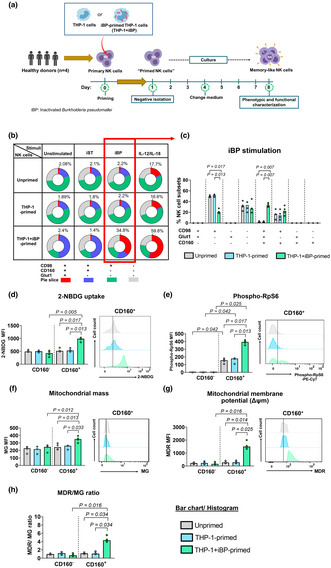

## Introduction

Immune responses are linked to extensive and rapid alteration in immune cell function, and it has become obvious that the activities of such cells depend on highly dynamic changes in cellular metabolism.^1^ Metabolic pathways are constantly rearranged to meet biosynthetic and bioenergetic demands of immune cells. It has emerged that cellular metabolism plays a critical role in the regulation of immune cell effector functions and immune signalling.^1^, ^2^ Recent studies have revealed more complex roles for metabolic pathways or metabolites in lymphocyte development and function.^3^, ^4^, ^5^ Naive T cells have limited biosynthetic demands and are in a quiescent state where oxidative phosphorylation (OXPHOS) is required for metabolic homeostasis.^3^ Glycolysis and tricarboxylic acid (TCA) cycle generate adenosine triphosphate (ATP) and metabolic intermediates to facilitate T cell differentiation, proliferation and cytokine production.^4^, ^5^ Memory T cells have a distinct metabolic pattern in which energetic requirements and survival are sustained by fatty acid oxidation and OXPHOS.^6^ T cell metabolism has been well characterised; however, cellular bioenergetics of natural killer (NK) cells remain to be elucidated.

Natural killer cells are traditionally considered a type of effector lymphocytes in innate immunity, which quickly respond to physiologically stressed cells such as infected cells and cancer cells.^7^ It is currently accepted that NK cells can acquire the hallmark features of adaptive immunity, owing to the discovery of long‐lived NK cells with non‐specific responses as well as antigen‐specific responses (memory NK cells or adaptive NK cells) upon re‐stimulation.^8^, ^9^, ^10^ Recent studies have illustrated that particular metabolic reprogramming is essential for functional responses of activated NK cells, including cytokine production and cytotoxicity.^11^, ^12^, ^13^ This metabolic rearrangement encompasses the upregulation of metabolic enzymes and nutrient transporters as well as an increase in mitochondrial mass, allowing enhanced biosynthetic capacity and ATP synthesis via increased glycolytic flux and OXPHOS.^13^ Adaptive NK cells have more metabolically active phenotypes than canonical NK cells, primarily manifested as heightened glycolysis and mitochondrial oxidative metabolism.^14^ Cytokine‐induced memory‐like NK cells exhibit a metabolic profile shifted towards the glycolytic pathway.^15^

The role of NK cell metabolism has been studied in several diseases including cancer, obesity and infection.^16^, ^17^ However, evidence of metabolic requirements of NK cells in response to infection is still sparse with cytomegalovirus (CMV) and Friend virus (FV) being the only pathogens studied so far.^14^, ^17^, ^18^, ^19^ In the context of antiviral responses against CMV, NK cell‐mediated control of mouse cytomegalovirus (MCMV) infection is dependent on glucose metabolism.^17^ NK cells also require glycolysis and OXPHOS to produce IFN‐γ and Granzyme B although each function can be controlled by a different mechanism.^12^, ^18^ Adaptive NK cells from human cytomegalovirus (HCMV)‐seropositive individuals display upregulation of multiple genes encoding components of the mitochondrial electron transport chain (ETC) and ATP synthase complex, and increased mitochondrial membrane potential.^14^ In addition, NK cells can reprogram their metabolic machinery by increasing glycolysis and mitochondrial metabolism and increasing nutrient uptake, including amino acids and iron following FV infection in a mouse model.^19^

Natural killer cells play a critical role in controlling intracellular bacterial infection^20^ and are emerging as an immune correlate of protection in acute melioidosis,^21^ a frequently overlooked tropical infection caused by the soil‐dwelling Gram‐negative bacterium *Burkholderia pseudomallei* (BP). Melioidosis is under‐reported globally with an estimated 165 000 human cases and annual 89 000 deaths.^22^ The disease affects vulnerable populations, and type 2 diabetes (T2DM) is a major risk factor.^23^ However, there is no licensed vaccine against this bacterial infection, which further exacerbates concerns of a possible emerging public health threat.^24^ A better knowledge of NK cell immunity in the context of bacterial infection might be a critical avenue to target NK cells for vaccine development against Gram‐negative bacterial infections.

Our previous study has demonstrated that NK cells require BP‐primed monocytes to acquire memory‐like properties, and we identified a novel human memory‐like NK cell population expressing CD160 in recovered melioidosis patients.^25^ However, NK cell metabolism and its importance for memory‐like NK cell response to bacterial infection remain scarce. Here, we investigated metabolic requirements for memory‐like NK cell generation and function in response to the Gram‐negative bacterium BP. We highlight metabolic similarities to memory T cells and identify metformin as a potential enhancer of memory‐like NK cell formation. This has implications for future vaccine design and development of therapeutics in the context of Gram‐negative bacterial infections.

## Results

### Memory‐like CD160^+^ NK cells from recovered melioidosis patients increase nutrient transporter expression upon BP re‐stimulation

**Figure 1:**
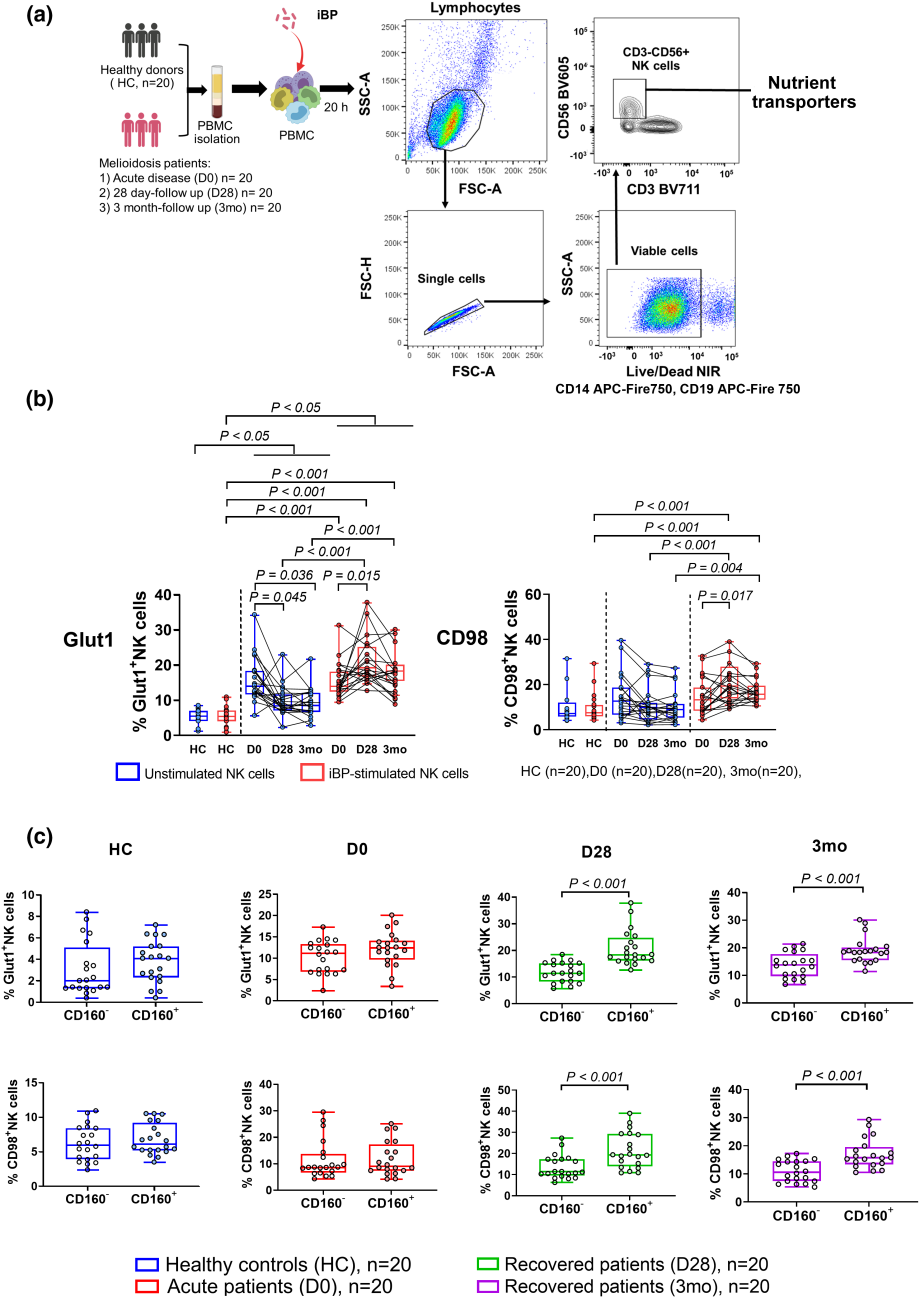
CD160^+^memory‐like NK cells from recovered melioidosis patients showed increased expression of amino acid and glucose transporters upon iBP stimulation. **(a)** NK cells were isolated from PBMC of healthy endemic controls (HC, *n* = 20) and melioidosis patients with acute disease (D0, *n* = 20) and 28‐day (D28, *n* = 20) and 3‐month (3mo, *n* = 20) follow‐up. NK cells were incubated in the absence or presence of inactivated BP (iBP) for 20 h and then subjected to analysis of nutrient transporter expression on NK cells. The gating strategy is shown in representative flow cytometry dot plots. Lymphocytes (FSC‐A, SSC‐A) were gated followed by a single cell gate (FSC‐A, FSC‐H) and a viable cell gate (Live/Dead neg/lo) excluding lineage markers CD14 and CD19. Bulk NK cells were identified as CD56^+^ and CD3^−^ within the CD14^−^/CD19^−^/viable cell gate and expression of NK cell nutrient transporters was identified within this gate. **(b)** The percentages of NK cells expressing Glut1 or CD98 cultured in the absence or presence of BP stimulation were determined by flow cytometry. **(c)** The percentage of Glut1‐ or CD98‐expressing NK cells within the CD160^−^ and CD160^+^ subsets upon iBP stimulation. Boxplots with whiskers represent 25th and 75th percentile boundaries in the bow with the individual plot and the median line within the box. Statistical significance between multiple independent groups (healthy donors and melioidosis patients) was determined using the Kruskal–Wallis test, followed by Dunn's test with the Benjamini–Hochberg method for multiple comparisons. The Friedman's test, followed by Dunn's method with the Benjamini–Hochberg method for multiple comparisons, was used to compare statistical differences between multiple dependent groups (acute and recovered melioidosis patients, *n* = 20 in each group). A two‐tailed Wilcoxon matched‐pairs signed‐rank test was used for comparing Glut1 and CD98 expressions in CD160^−^ vs. CD160^+^ NK cells from healthy donors (*n* = 20) and melioidosis patients (D0, D28 and 3mo, *n* = 20 in each group). A *P*‐value of < 0.05 was considered statistically significant.

We have previously identified a population of CD160^+^ memory‐like NK cells in recovered melioidosis patients with the total number of NK cells comparable between healthy individuals and melioidosis patients.^25^ Using the same cohort from Northeast Thailand, composed of 77% patients with T2DM comorbidity, we asked whether the expression of nutrient transporters CD98 (amino acid transporter) and GLUT1 (glucose transporter) on NK cells correlates with the expression of the memory‐like NK cell marker CD160 in melioidosis patients during acute disease (D0), 28‐day (D28) and 3‐month (3mo) follow‐up (Figure 1a).

Unstimulated NK cells from acute patients (D0) had significantly increased Glut1 expression compared to healthy individuals and recovered patients (D28 and 3mo) (*P* < 0.05, for all comparisons). Upon stimulation of PBMC with inactivated BP (iBP) *ex vivo*, NK cells upregulated the expression of nutrient transporters with a median increase of 19.36% (IQR 16.84–25.33%) in Glut1 and 19.32% (IQR 13.93–27.95%) in CD98 at 28‐day follow‐up, compared to the unstimulated condition. Expression of nutrient transporters in stimulated cells slightly dropped at the 3‐month follow‐up time point but remained significantly elevated compared to the unstimulated control (Figure 1b). iBP stimulation induced CD160^+^NK cells to express higher levels of Glut1 and CD98 than CD160^−^ NK cells in recovered melioidosis patients at 28‐day and 3‐month follow‐up time points (Figure 1c), indicating that CD160^+^ NK cells from recovered melioidosis patients are metabolically more active.

### BP‐specific memory‐like NK cells generated *in vitro* are metabolically primed

**Figure 2:**
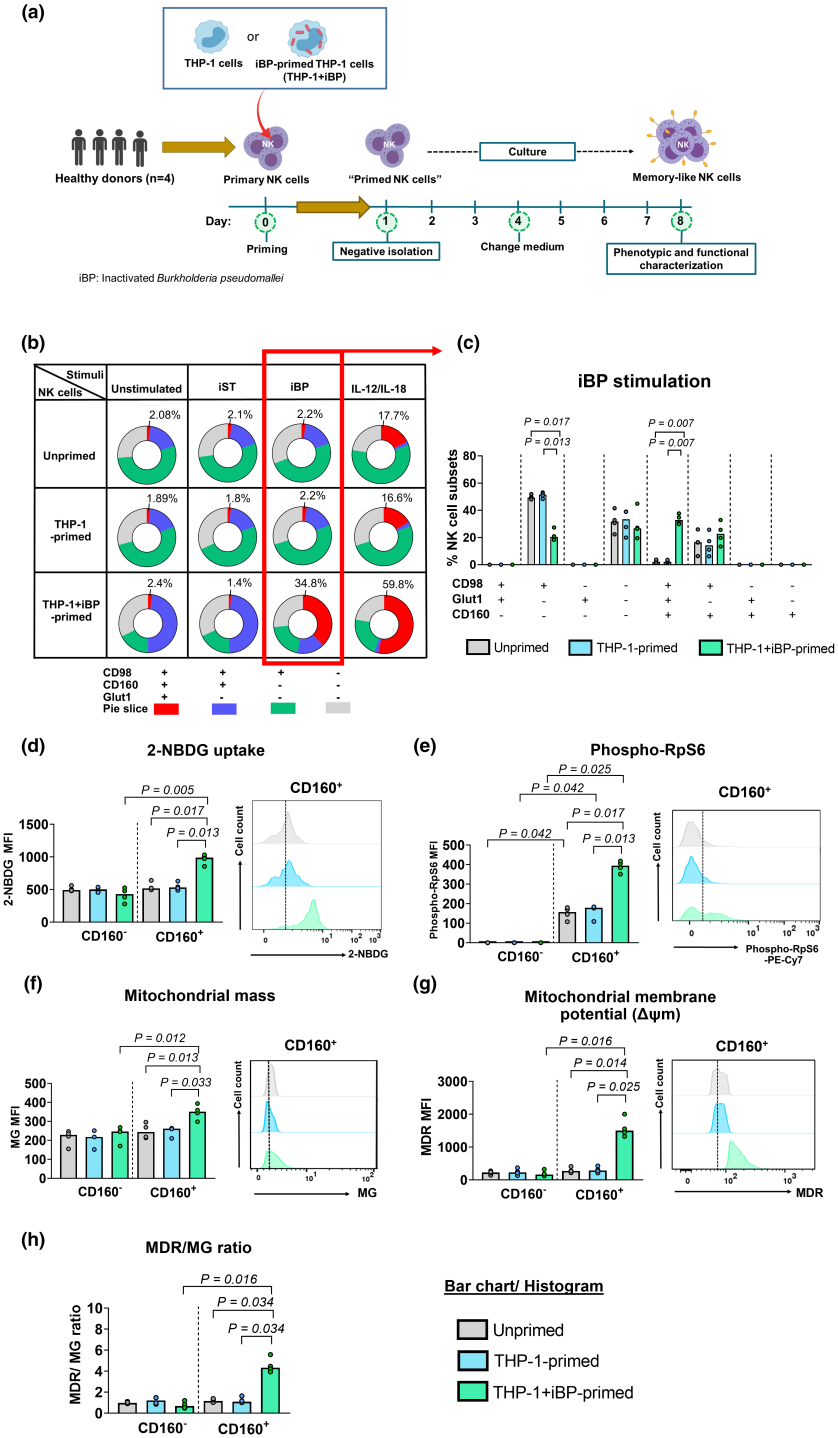
Metabolic characteristics of memory‐like NK cells generated *in vitro*. **(a)** Schematic of experiments for NK cell memory assay (created with Biorender.com and adapted from our previous study^25^). Primary NK cells isolated from PBMCs of healthy donors (*n* = 4) were primed with inactivated BP‐primed THP‐1 cells (THP‐1 + iBP), unprimed THP‐1, or left them unprimed for 24 h (days 0–1). Primed NK cells were purified by magnetic isolation and subsequently cultured for 7 days (days 1–8) in NK expansion medium. On day 7, unprimed‐, THP‐1‐primed or THP‐1 + iBP‐primed NK cells were stimulated with inactivated *Burkholderia pseudomallei* (iBP), inactivated *Salmonella* enterica serovar Typhi (iST) or IL‐12/IL‐18 for 18 h. Metabolic features of NK cells were determined by flow cytometry. **(b)** The percentage of NK cells co‐expressing CD98, CD160 and Glut1. The pie chart summarises the data from four healthy donors with three technical replicates measured per donor (three independent experiments). Each slice presents the subsets of NK cell expressing a given combination of molecules within the unprimed and primed NK cell populations. The median percentage of responding cells with the co‐expression of three phenotypes is given above each pie chart. **(c)** Bar graph showing a detailed breakdown of the proportion of cells co‐expressing CD98, Glut1 and CD160 upon iBP stimulation (corresponding to data highlighted in red in **b**). **(d–h)** Metabolic features of unprimed and primed NK cells in CD160^−^ and CD160^+^ subsets upon iBP stimulation: **(d)** glucose uptake (glucose analogue 2‐NBDG), **(e)** mTOR activation (phosphorylated RpS6), **(f)** mitochondrial mass, **(g)** mitochondrial membrane potential (Δψm) and **(h)** mitochondrial membrane potential normalised by mass (MitoTracker Deep Red (MDR)/ MitoTracker Green (MG)) in median fluorescence intensity (MFI). Data from four individual donors are presented in bar graphs. Each data point represents the median of three technical replicates (independent experiments) for one healthy donor. The medians of the technical replicates were used for statistical testing and graphical presentation. Statistical analyses were performed using a two‐tailed Wilcoxon matched‐pairs signed‐rank test between each priming condition in CD160^−^ vs. CD160^+^ NK cells, and the Friedman's test, followed by the Dunn's test with the Benjamini–Hochberg method for comparing different priming conditions in either CD160^−^ or CD160^+^ NK cells. Only *P‐*values for statistically significant (*P* < 0.05) comparisons are shown on the graphs.

To further dissect the metabolic characteristics of BP‐specific memory‐like NK cells, we used our previously established *in vitro* assay for generation of memory‐like NK cells through priming with inactivated BP (iBP)‐primed THP‐1 cells (THP‐1 + iBP) (Figure 2a). THP‐1 + iBP‐primed NK cells had BP‐specific memory‐like properties characterised by heightened polyfunctional responses (CD107a, IFN‐g) to BP but not unrelated pathogens and increased cytotoxicity compared to unprimed and THP‐1 primed NK cells, as described in our previous study.^25^

To corroborate our *in vivo* findings, we first explored the co‐expression of nutrient transporters and CD160 on *in vitro* generated memory‐like NK cells (THP‐1 + iBP‐primed NK cells) upon re‐stimulation with iBP, an unrelated bacterial stimulus (inactivated *Salmonella typhi*, iST) as well as cytokines (IL‐12/IL‐18). IL‐12 and IL‐18 have been previously reported to induce metabolic activity of NK cells, such as increased expression of nutrient transporters.^15^, ^26^, ^27^ Indeed, THP‐1 + iBP‐primed NK cells markedly increased the proportion of cells co‐expressing CD160, CD98 and Glut1 when stimulated with iBP or IL‐12/IL‐18, compared to controls (unprimed or THP‐1‐primed NK cells) (Figure 2b and c, Supplementary figure 3b). In contrast, there was no change in co‐expression of these markers in THP‐1 + iBP‐primed NK cells when stimulated with iST (Supplementary figure 3b). This is in line with our previous observation that the CD160^+^ subset contains the BP‐specific memory‐like NK cells. Concomitantly with an increased expression of the glucose transporter Glut1, CD160^+^ memory‐like NK cells (THP‐1 + iBP‐primed) showed a significant increase in glucose uptake (2‐NBDG) upon iBP and IL‐12/IL‐18 stimulation compared to the respective controls (unprimed or THP‐1 primed) (Figure 2d, Supplementary figure 4b). No differences were observed in neither the CD160^−^ NK cell subset nor response to an unrelated bacterial stimulus (iST) (Supplementary figure 4c).

The mechanistic target of rapamycin (mTOR) signalling pathway is a central metabolic regulator in NK cells^28^ and has been shown to be activated in CMV‐induced adaptive NK cells.^29^ To study the activation of mTORC1, we measured phosphorylation of its downstream target Ribosomal Protein S6 (RPS6). First, CD160^+^ memory‐like NK cells (THP‐1 + iBP‐primed) were characterised by increased phosphorylation of RpS6 in response to any stimuli (iBP, iST and IL‐12/IL‐18) compared to the CD160^−^ NK cell subset (THP‐1 + iBP‐primed), and this was independent of priming condition. However, only the CD160^+^ memory‐like NK cells displayed significantly higher mTOR activation in response to iBP and cytokine stimulation compared to the control (THP‐1 primed) (*P* < 0.05 for all comparisons) (Figure 2e, Supplementary figure 4b), which was not observed in the presence of iST (Supplementary figure 4c).

We further determined mitochondrial mass and membrane potential (Δψm) using MitoTracker Green (MG) and MitoTracker Deep Red (MDR), respectively. We found that THP‐1 + iBP‐primed NK cells have increased levels of mitochondrial mass and membrane potential (absolute and normalised to mitochondrial mass) compared to unprimed or THP‐1 primed NK cells upon any re‐stimulation condition (iBP, iST or IL‐12/IL‐18) (*P* < 0.05 for all comparisons). Again, this was specifically associated with the CD160 expressing NK cell subset (Figure 2f–h, Supplementary figure 4) and suggests that CD160^+^ memory‐like NK cells undergo changes in mitochondrial quality.

Overall, these results show that CD160^+^ memory‐like NK cells are metabolically primed.

### Oxidative phosphorylation is required for BP‐specific memory‐like NK cell function

**Figure 3:**
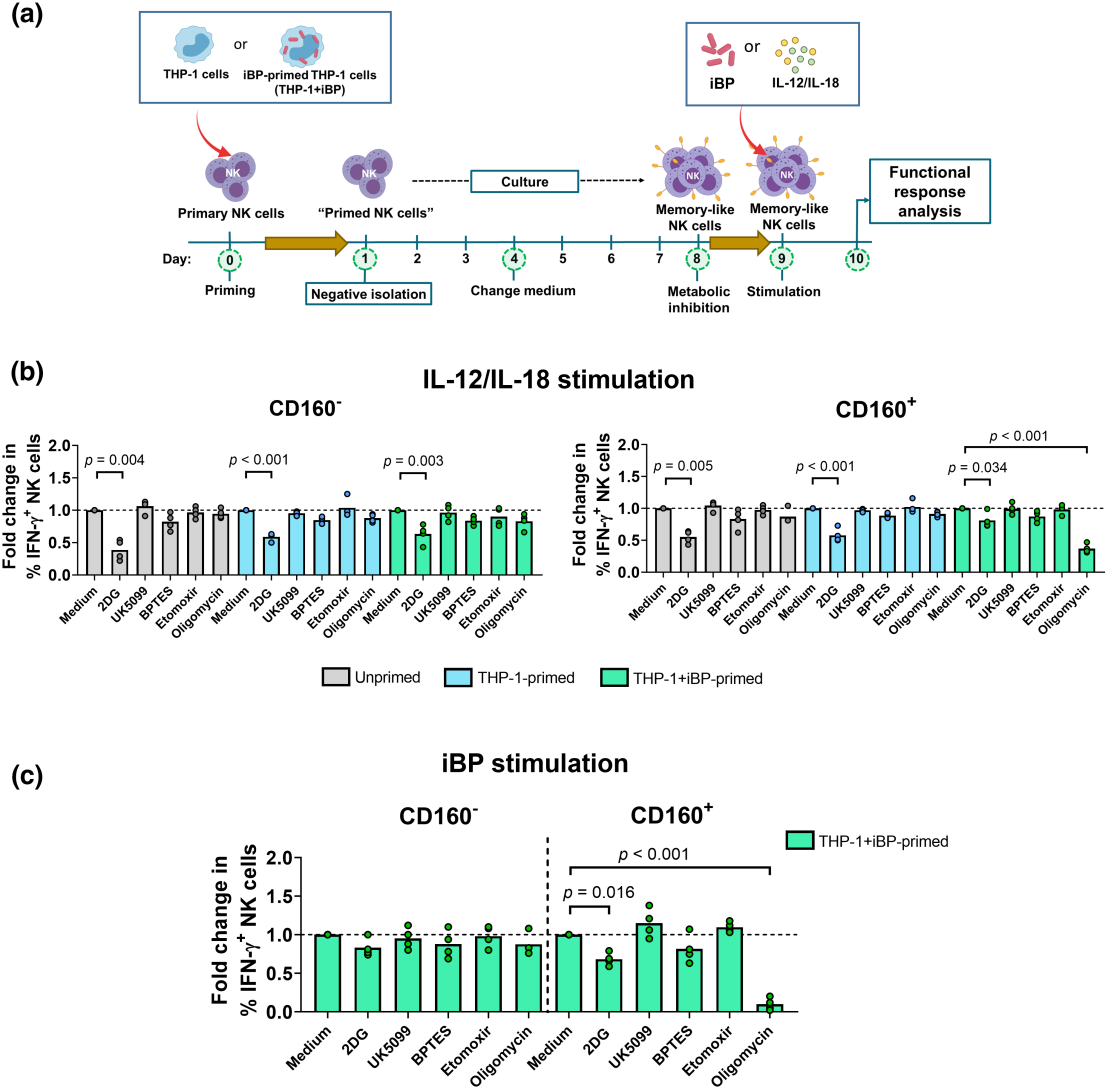
Metabolic requirement for BP‐specific memory‐like NK cell function. **(a)** The NK cell memory assay was performed to generate CD160^+^ memory‐like NK cells *in vitro*. Unprimed‐, THP‐1‐primed or THP‐1 + iBP‐primed NK cells were treated with the following metabolic regulators: 2‐DG, UK5099, BPTES, etomoxir or oligomycin A at 8‐day post‐priming and then stimulated with iBP, or IL‐12/IL‐18 for 18 h. **(b)** The fold change of IFN‐γ production (the percentage of IFN‐γ^+^ NK cells in the CD160^−^ and CD160^+^ subsets) upon **(b)** IL‐12/IL‐18 stimulation of unprimed‐, THP‐1‐primed‐ or THP‐1 + iBP‐primed NK cells or **(c)** iBP stimulation of THP‐1 + iBP‐primed NK cells in the CD160^−^ and CD160^+^ subsets the presence of metabolic inhibitor treatment relative to the absence of metabolic inhibitor treatment. The horizontal dashed line on each graph represents a fold change of 1, where expression levels on NK cells in the absence and the presence of metabolic inhibitors are equal. Data from four individual donors are presented in bar graphs. Each data point represents the median of three technical replicates (independent experiments) for each healthy donor. The medians of the technical replicates were used for statistical testing and graphical presentation. Statistical analysis was performed using the Friedman's test, followed by the Dunn's test with the Benjamini–Hochberg method for multiple comparisons, and only *P*‐values for statistically significant (*P* < 0.05) comparisons are shown on the graphs.

We next examined the metabolic dependency of memory‐like NK cells in response to iBP and IL‐12/IL‐18 stimulation. The following metabolic inhibitors were used to treat unprimed and primed NK cells at day 8 post‐priming (Figure 3a): 2‐deoxy‐d‐glucose (2‐DG, a glucose analogue that inhibits glycolysis), UK5099 (an inhibitor of the mitochondrial pyruvate carrier), bis‐2‐(5‐phenylacetamido‐1,3,4‐thiadiazol‐2‐yl) ethyl sulfide (BPTES, an inhibitor of glutaminase), etomoxir (an inhibitor of fatty acid oxidation) and oligomycin A (an inhibitor of mitochondrial ATP synthase and uncoupler of OXPHOS). The IFN‐γ response to IL‐12/IL‐18 stimulation was dampened upon glycolysis inhibition (2‐DG‐treated) in all NK cells, either CD160^−^ or CD160^+^, primed or unprimed condition. Intriguingly, the inhibition of OXPHOS was sufficient to significantly reduce the IFN‐γ response in THP‐1 + iBP‐primed NK cells in only the CD160^+^ subset upon IL‐12/IL‐18 stimulation, compared to non‐treated condition (*P* < 0.05) (Figure 3b). Similar to cytokine stimulation, iBP stimulation reduced IFN‐γ response in THP‐1 + iBP‐primed NK cells expressing CD160 when glycolysis and OXPHOS were inhibited (Figure 3c). These results indicate that CD160^+^ NK cells remarkably require OXPHOS to mount memory‐like response.

### Development of CD160^+^ memory‐like NK cells *in vitro* requires fatty acid oxidation, oxidative phosphorylation and is amplified by known enhancers of autophagy

**Figure 4:**
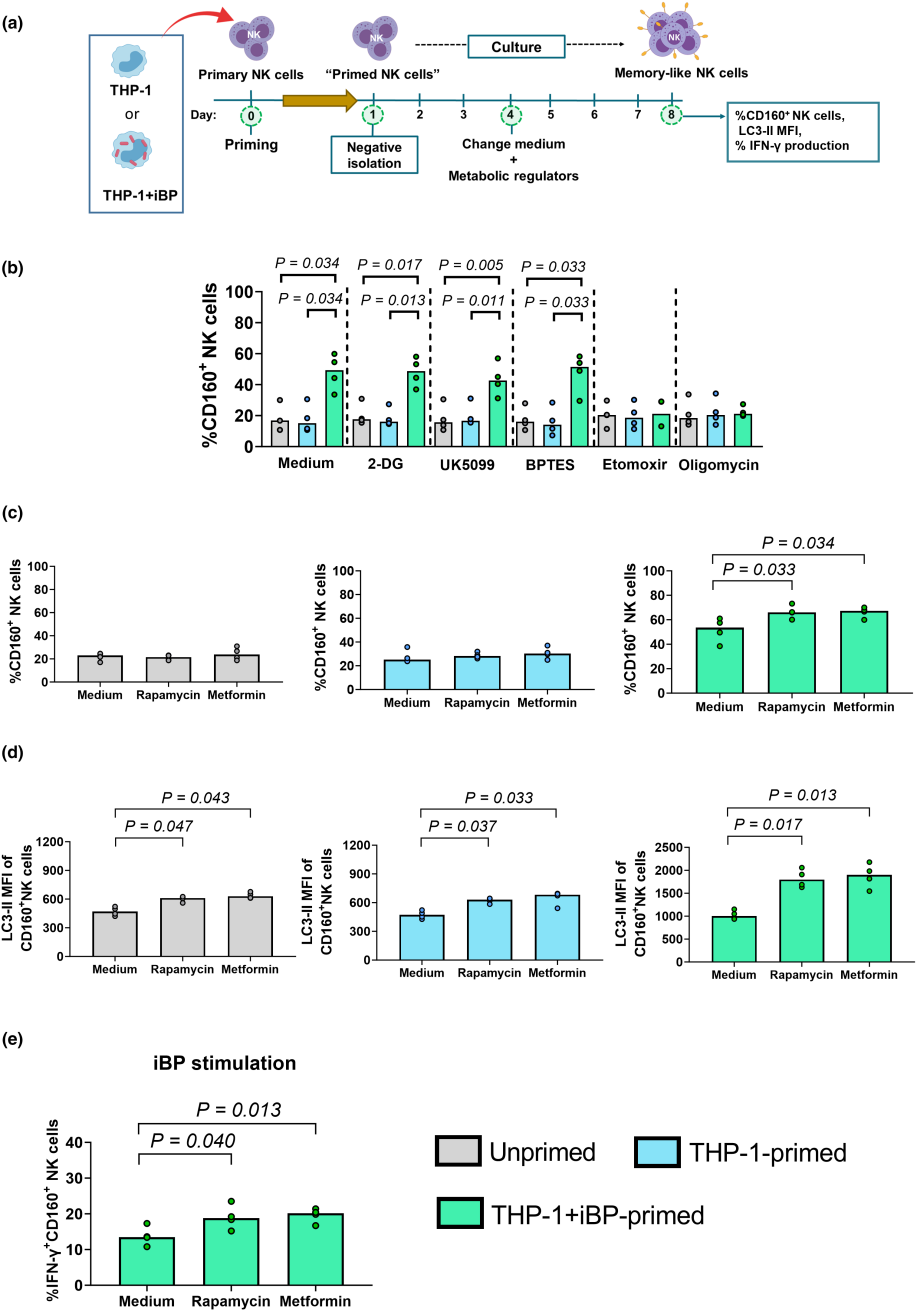
Metabolic requirement for generation of BP‐specific memory‐like NK cells. **(a)** The NK cell memory assay was performed to generate CD160^+^ memory‐like NK cells *in vitro*. Unprimed‐, THP‐1‐primed or THP‐1 + iBP‐primed NK cells were treated with the following metabolic regulators: 2‐DG, UK5099, BPTES, etomoxir, oligomycin A, metformin or rapamycin at 4‐day post‐priming. A frequency of CD160^+^ NK cells and autophagic activity were assessed. **(b)** Metabolic dependency of unprimed and primed NK cells on expression of the NK memory marker CD160. **(c)** Effects of metformin and rapamycin treatment on the relative frequency of in CD160^+^ NK cells and **(d)** LC3‐II MFI in CD160^+^ NK cells cultured under unprimed‐, THP‐1‐primed or THP‐1 + iBP conditions. **(e)** The percentage of IFN‐γ production in CD160^+^ THP‐1 + iBP‐primed NK cells in the absence or presence of metformin and rapamycin, respectively. Data from four individual donors are presented in bar graphs. Each data point represents the median of three technical replicates (independent experiments) for one healthy donor. The medians of the technical replicates were used for statistical testing and graphical presentation. Statistical analysis was performed using the Friedman's test, followed by the Dunn's test with the Benjamini–Hochberg method for multiple comparisons, and only *P*‐values for statistically significant (*P* < 0.05) comparisons are shown on the graphs.

Finally, we assessed the metabolic basis for the expansion of BP‐specific CD160^+^ memory‐like NK cells using the same metabolic inhibitors as described above. NK cells primed with THP‐1 + iBP, THP‐1 alone or unprimed were treated with these inhibitors on day 4 of culture, and their effect on the formation of CD160^+^ memory‐like NK cells was assessed (Figure 4a). The inhibition of fatty acid oxidation and OXPHOS (by etomoxir and oligomycin A treatment, respectively) completely blocked the enrichment of the CD160^+^ memory‐like population in THP‐1 + iBP‐primed NK cells, whereas the other inhibitors did not have an effect (Figure 4b). Of note, none of the metabolic inhibitors had an effect on the NK cell viability and the absolute number of live NK cells (Supplementary figure 5).

Autophagy is important in memory T cell formation and maintenance.^30^ Because of the functional and metabolic similarities observed between memory T cells and memory‐like NK cells, we hypothesised that autophagy is also an important process implicated in BP‐specific CD160^+^ memory‐like NK cells. It is known that inhibition of active mTOR signalling by activation of AMPK can induce autophagy.^31^ In order to address whether enhanced autophagy could promote the generation of memory‐like NK cells, we treated THP‐1 + iBP‐primed NK cells with the known AMPK activator and anti‐diabetic drug metformin^32^ as well as the mTOR inhibitor rapamycin^33^ at 4‐day post‐priming.

THP‐1 + iBP‐primed NK cells showed more than 30% increase in the frequency of CD160^+^ NK cells upon metformin and rapamycin treatment compared to the untreated culture (Figure 4c). Furthermore, both autophagy enhancers remarkably increased the expression of LC3‐II (a standard marker for autophagosomes^34^) (more than 80%) and IFN‐γ response upon iBP stimulation in CD160^+^ memory‐like NK cells (THP‐1 + iBP‐primed) (Figure 4d and e).

These results illustrate that the formation of CD160^+^ memory‐like NK cells is dependent on fatty acid oxidation, OXHOS and enhanced by autophagy inducers.

## Discussion

Bacteria are the cause of numerous human diseases worldwide. Understanding host immune responses as well as potential anti‐bacterial immunity are urgently required for combating bacterial infections that still cause public health issues without appropriate therapeutics or vaccinations such as melioidosis, a severe bacterial infection caused by the Gram‐negative bacterium BP. Cellular metabolism is a prerequisite for the normal function of immune cells in response to infectious disease.^35^ The study of immunometabolism is likely to reveal the intricate relationship between immune function and key metabolic pathways, which is essential for developing novel therapeutics and vaccination approaches. We aim to understand cellular metabolic processes underlying adaptive responses and the formation of memory‐like NK cells in melioidosis, which will inform vaccine design and further illuminate the importance NK memory in bacterial infections. Our previous study has revealed that CD160^+^ memory‐like NK cells develop upon melioidosis infection.^25^

It has long been recognised that NK cell can display features of immunological memory with more specific innate recognition in different infections, with the most prominent model being CMV infection.^36^ HCMV‐driven NK cell memory is mediated by unique subset of NK cells that co‐express CD94/NKG2C and CD57, proliferate after HCMV infection and persist for months.^37^, ^38^ This is in line with our previous study showing that CD160^+^ memory‐like NK cells characteristically co‐express increased CD57 and NKp30, and develop upon melioidosis infection with heightened responses maintained at least 3‐month post‐hospital admission.^25^ In contrast to NKG2C^+^ adaptive NK cells in HCMV infection, BP‐elicited memory‐like NK cells are highly sensitive to innate cytokines, such as IL‐12 and IL‐18, owing to upregulation of IL‐12/IL‐18 receptors and T‐bet.^25^, ^39^, ^40^ BP antigen‐primed monocytes and CD160 expression are also required for induction of BP‐specific NK cell memory,^25^ although no specific BP antigen recognised by NK cells has been identified yet. Specific recognition of distinct pathogens via NK cell receptors may be driven by avidity selection of NK cells, resulting in a shift of the receptor repertoire to increase the number of antigen‐specific NK cells with memory characteristics.^41^ This is evidenced by preferential expansion of NK cell subsets with highest avidity for MCMV antigens and epigenetic imprinting on the NK cell repertoire in HCMV infection.^41^, ^42^ However, the mechanism of antigen‐specific memory NK cell formation in other infections is a matter of debate.

In this study, we investigate the metabolic requirements of these memory‐like NK cells using our established *in vitro* NK cell memory assay, as well as clinical samples from a Thai melioidosis cohort. Our findings illustrate that the expression of nutrient transporters is correlated with the BP‐specific NK cell memory marker CD160 upon stimulation. Previous studies have shown that the upregulation of nutrient transporters on NK cells can be induced upon stimulation.^26^, ^43^, ^44^ Cytokine‐induced memory‐like (CIML) NK cells exhibit increased glucose uptake and expression of Glut1 and CD98 in response to IL‐12/15/18 stimulation.^15^ We also found distinct co‐expression patterns of Glut1 and CD98 on BP‐specific memory‐like NK cells, which is associated with CD160 expression and RpS6 phosphorylation. One study has illustrated that CD160 positively regulates glucose metabolism via the PI3K/AKT/mTOR signalling pathway and its expression is associated with enhanced expression of the glucose transporter Glut1.^45^ In addition, the increased expression of amino acid transporter CD98/LAT1 can induce the activation of the mTOR signalling pathway, which contributes to the acquisition of NK cell effector function.^26^

We show changes in mitochondrial quality of BP‐elicited memory‐like NK cells including increased mitochondrial mass and membrane potential. Previous studies have shown that cytokine‐stimulated NK cells and CIML NK cells are prone to increase mitochondrial mass^13^, ^15^, ^44^ and the CD56^bright^ NK cell subset also showed increased mitochondrial membrane potential.^44^ Greater mitochondrial membrane potential along with higher rates of OXPHOS was previously observed in adaptive compared to canonical NK cells.^14^

The inhibition of metabolic pathways in our experimental setting showed that OXPHOS is uniquely required for BP‐elicited memory‐like NK cell responses. Similarly, the inhibition of OXPHOS and the Sterol regulatory element‐binding proteins (SREBPs) transcription factor has been shown to prevent the enhanced functionality of CIML NK cells in response to IL‐12/15/18 stimulation.^11^, ^46^ In addition, HCMV‐adaptive NK cells have the ability to upregulate glycolysis and OXHOS upon activation.^14^ It is possible that memory‐like NK cells undergo distinct modes of epigenetic changes, accompanying functional modifications, as well as metabolic and transcriptional reprogramming.^47^ Whether bacteria‐induced memory‐like NK cells have a particular epigenetic modification contributing to cellular bioenergetics still needs to be investigated.

We further provide evidence that fatty acid oxidation and OXPHOS are essential for the formation of CD160^+^ memory‐like NK cells. This is in line with the metabolic requirement of memory T cells, which mainly rely on fatty acid oxidation and OXPHOS.^48^ In metabolically restricting conditions, we found that the formation of CD160^+^ memory‐like NK cells was remarkably suppressed when fatty acid oxidation and OXPHOS are inhibited. Recently, gene expression analyses of HCMV‐adaptive NK cells have elucidated the upregulation of lipid metabolism genes.^14^ Autophagy is able to regulate lipid metabolism through the degradation of intracellular lipid stores.^49^ However, further experiments would be needed to explain whether lipid substrates for fatty acid oxidation during the formation of CD160^+^ memory‐like NK cells are generated through autophagy.

We also demonstrate that metformin treatment, known to activate AMPK, or inhibition of mTOR by rapamycin treatment enhanced the generation of CD160^+^ memory‐like NK cells. Autophagic activity is mainly controlled by AMPK and mTOR regulators.^50^ Activation of AMPK can enhance autophagic activity via its ability to actively suppress mTOR signalling.^31^ It has been previously shown that treatment with either metformin or rapamycin enhanced the formation of lymphocytic choriomeningitis virus (LCMV)‐specific memory CD8^+^ T cells and MCMV‐specific adaptive NK cells during the contraction phase after viral infection.^33^, ^34^, ^51^ Metformin is one of the most widely prescribed drugs for the treatment of T2DM.^52^ Moreover, metformin treatment has the potential to regulate immune cells irrespective of current infections and likely improve responses to future pathogens.^53^ Metformin may be a promising drug for improving vaccine‐induced NK cell memory and immune responses to natural infection in people with chronic metabolic diseases and the elderly.

In conclusion, this study demonstrates the impact of cellular metabolic demands on human memory‐like NK cell formation and response to bacterial infection. These findings would provide advantages for future monitoring of next‐generation vaccines in which NK cell could be metabolically reprogrammed to induce memory and memory‐like responses, and highlight the necessity to continue exploring the mechanistic basis of NK cell metabolism to find novel and reliable approaches of profiling NK cell energetics and functional fates across infectious diseases.

## Limitations of study

There are several limitations to this study which are worth noting. First, we were limited regarding the number of melioidosis patients because of loss to follow‐up and feasibility of recruitment. Second, replication for other bacterial infections is required to recapitulate the relationship between NK cell metabolism, functional fate and phenotype across different infectious diseases. Third, the increase in LC3II expression simply reflects the accumulation of autophagosomes in autophagy but does not indicate the autophagic flux. Further studies exploring the relevance of autophagic degradation in development of CD160^+^ memory‐like NK cells are underway. Finally, approximately 70% of analysed patients had diabetes, and the number of people without diabetes was not sufficient to perform statistical analysis by diabetes status. Experimental evaluation of how this may affect memory‐like NK cell metabolism in melioidosis needs to be further investigated.

## Methods

### Ethics statement

Human studies and consent forms were reviewed and approved by the Ethics committees of the Faculty of Tropical Medicine, Mahidol University (MUTM 2021‐033‐01), Mukdahan Hospital (MEC 07/64) and the University of Oxford (REC 21/YH/0206). The study was conducted according to the principles of the Declaration of Helsinki (2008), the International Conference on Harmonisation (ICH) and Good Clinical Practice (GCP) guidelines. All volunteers provided written informed consent to participate in this project.

### Study design and subjects

Thirty healthy donors and 20 melioidosis patients were recruited at Mukdahan Hospital, Mukdahan, Thailand. Healthy donors were enrolled at the hospital's blood donation clinic. Inclusion criteria for the healthy cohort were male or female of age ≥ 18 years. Exclusion criteria were pregnancy or delivery in the past 9 months, weight of less than 40 kg or greater than 136 kg, previous history of melioidosis, recent illness, any chronic medical condition or medications and any organ failure (such as cirrhosis), any immune system deficiency, vaccination within the past 6 weeks, use of any immune‐modifying agents or any anti‐inflammatory medications or cell depletion biological agents in the past week, infectious symptoms in the past 2 weeks, vigorous exercise in the past 24 h, or alcohol use in the past 24 h. For the melioidosis cohort, inclusion criteria were female or male patients aged 18 years or older with *B. pseudomallei* culture‐confirmed melioidosis from any clinical specimens taken within 24 h after hospital admission. Exclusion criteria were receiving palliative care, incarceration or pregnancy. *B. pseudomallei* was culture‐confirmed by latex agglutination and biochemical tests^54^ at the Microbiology laboratory of the hospital and further confirmed by Matrix‐Assisted Laser Desorption Ionisation Mass Spectrometry (MALDI‐TOF MS) as previously described.^55^

Whole blood samples were collected from healthy donors and melioidosis patients on the day of recruitment (defined as day 0). Blood samples were further obtained from melioidosis patients at 28‐day and 3‐month follow‐up time points. Clinical data were recorded by extracting information from medical records. Mortality data were collected from follow‐up phone calls for 28 days and hospital mortality records.

### PBMC isolation

Peripheral blood mononuclear cells (PBMCs) were obtained from 20 mL of heparinised blood within 3 h of sample collection. Briefly, blood was diluted 1:1 in R10 media: RPMI 1640 (Invitrogen, CA, USA) supplemented with 10% heat‐inactivated foetal bovine serum (FBS) (Himedia, Mumbai, India), 1 mm Penicillin/Streptomycin (Sigma, St. Louis, MO, USA) and 2 mm GLutaMAX (Invitrogen) prior to transfer to 50 mL SepMate tubes (STEMCELL Technologies, Vancouver, Canada) containing 15 mL of Lymphoprep (Axis Shield, Oslo, Norway). PBMCs were isolated by density gradients centrifugation using Lymphoprep. PBMCs were cryopreserved in FBS containing 10% dimethyl sulfoxide (DMSO) (Sigma) in liquid nitrogen until use.

### NK cell isolation from PBMC

Primary NK cells were isolated from at least 5 × 10^6^ previously cryopreserved PBMCs using EasySep Human NK cell Enrichment Kit and a Purple EasySep Magnet according to manufacturers' instruction (STEMCELL Technologies). The purity of NK cells assessed by flow cytometry was > 95% of CD3^−^CD56^+^ cells.

### Cell lines and culture conditions

The human monocytic leukaemia cell lines (THP‐1, ATCC TIB‐202) were cultured in R10 media at 37°C with 5% CO_2_. For enrichment of isolated primary NK cells, the cells were cultivated in NK MACs medium (Miltenyi Biotec, Bergisch‐Gladbach, Germany) supplemented with 10% human AB serum (Sigma), 1 mm Penicillin/Streptomycin (Sigma), 5 ng mL^−1^ of rhIL‐2 and rhIL‐15 (Biolegend, San Deigo, USA) at 37°C with 5% CO_2_ for 7–10 days.

### Preparation of inactivated bacteria

Bacterial culture was performed in a BSL‐3 laboratory. *Burkholderia pseudomallei* (BP) strain K96243 and *Salmonella enterica* serovar Typhi (ST) strain CT18 were cultured in Luria broth (Oxoid, Hampshire, UK) with shaking at 250 rpm in a 37°C incubator for 18 h. The bacteria were collected with a loop, suspended and washed twice in 1 mL of PBS. The bacterial cells were diluted in 1× PBS to the desired concentration. Bacterial colony count was performed to determine the number of bacteria. Bacteria were resuspended at 10^9^ CFU mL^−1^ in 0.5% paraformaldehyde (PFA) in 1× PBS and fixed at room temperature for 20 min. PFA‐fixed bacteria were washed twice and resuspended in 1× PBS. Antigen sterility was confirmed by bacterial colony count. The PFA‐fixed bacteria were stored at −20°C until use.

### NK cell memory assay

The NK cell memory assay was performed as previously described.^25^ Briefly, THP‐1 monocytic cells resuspended in 1 mL of R10 were plated at 4 × 10^5^ live cells well^−1^ into 24‐well flat bottom plates. THP‐1 cells were primed with formalin‐fixed *B. pseudomallei* (BP) at a multiplicity of infections per cell (MOI) of 100 at 37°C with 5% CO_2_ for 24 h. To prime NK cells on day 0, isolated primary NK cells from healthy donors (*n* = 4) were resuspended in R10 at 6 × 10^5^ live cells and co‐cultured with BP‐primed THP‐1 cells (THP‐1 + BP) at 37°C with 5% CO_2_ for 24 h. Primed NK cells were subsequently purified by using EasySep human NK cell enrichment kit (STEMCELL Technologies) as described above. Primed NK cells resuspended in NK MACs medium (Miltenyi Biotec) supplemented with 10% human AB serum (Sigma), 1 mm Penicillin/Streptomycin (Sigma), 5 ng mL^−1^ of rhIL‐2 and rhIL‐15 (Biolegend) were plated at 3.5 × 10^5^ live cells/well into 24‐well flat bottom plates at 37°C with 5% CO_2_ for 7 days for further experiments. Culture medium was refreshed on day 4.

### Flow cytometry staining

For extracellular staining (ECS), cells were washed with 1× PBS and then resuspended in FACS buffer (Biolegend). Cells were incubated with near‐infrared live/dead fixable stain (Invitrogen, CA, USA) and fluorochrome‐conjugated primary human‐specific monoclonal antibodies in the presence of human FcR blocking reagent (Miltenyi Biotec) at 4°C for 20 min in the dark. Cells were washed twice with FACS buffer and fixed with IC fixation buffer (eBioscience) before acquiring on a flow cytometer. For intracellular staining (ICS), Brefeldin A (BD Biosciences Franklin Lakes, NJ, USA) was added at final dilution of 1:1000 to the cells 4 h prior to staining, following the manufacturer's recommendations. The cells were subjected to ECS and then washed and fixed with 1× fixation/permeabilisation solution (BD Biosciences, for cytokine staining) or 1 × True Nuclear Fix concentrate (Biolegend, for phospho‐protein staining) at 4°C for 20 min, washed with 1× permeabilisation buffer (BD Biosciences, for cytokine staining) or 1× True Nuclear Perm buffer (Biolegend, for phospho‐protein staining) followed by incubation with fluorochrome‐conjugated human‐specific monoclonal antibodies in the presence of human FcR blocking reagent at 4°C for 20 min in the dark. After washing with permeabilisation buffer, the sample was resuspended in FACS staining buffer and acquired immediately or stored at 4°C in the dark for up to 24 h prior to acquisition on a MACSQuant Analyzer 10 (Miltenyi Biotec) and FACAriaIII (BD bioscience). Data analysis was performed with a FlowJo software, version 10 (TreeStar, SanCarlos, CA, USA). Fluorochrome‐labelled monoclonal antibodies (mAbs) labelled with fluorochrome are shown in Supplementary table 5.

### Cell stimulation assay

After a 7‐day culture, unprimed or primed NK cells resuspended in R10 were plated at 200 000 cell/well in 96‐well round‐bottom plates in the presence and absence of PFA‐fixed bacteria (BP or ST) at final MOI of 20, or incubated with rIL12 plus rhIL‐18 (Biolegend) at a final concentration of 10 ng mL^−1^ at 37°C with 5% CO_2_ for 18 h. After stimulation, cells were washed with 1× PBS and subjected to flow cytometry staining (ECS and ICS) for analysis of NK cell characteristics.

For stimulation of PBMCs isolated from healthy donors and melioidosis patients in a Thai cohort, cells resuspended in R10 were plated at 5 × 10^5^ live cells per well into 96‐well round‐bottom plates and stimulated with inactivated BP at MOI of 100. Plates were placed at 37°C with 5% CO_2_ for 20 h and subjected to flow cytometry staining as described above.

### Metabolic assay

Mitochondrial mass and mitochondrial polarisation were assessed by performing the NK cell differentiation assay, as described above. After 7‐day cultivation, unprimed and primed NK cells resuspended in R10 were plated at 250 000 cell/well in 96‐well round‐bottom plates and stained with MIitoTracker Green FM (Invitrogen) and MitoTracker Deep Red (Invitrogen) at final concentration of 12 nm. Cells were incubated at 37°C with 5% CO_2_ for 30 min prior to flow cytometry staining and analysis.

To assess the uptake of the fluorescent glucose analogue 2‐NBDG (2‐(N‐(7‐Nitrobenz‐2‐oxa‐1,3‐diazol‐4‐yl)Amino)‐2‐Deoxyglucose), unprimed and primed NK cells resuspended in R10 were plated at 250 000 cell/well in 96‐well round‐bottom plates in the absence or presence of formalin‐fixed bacteria at final MOI of 20, or rhIL‐12 plus rhIL‐18 (Biolegend) at final concentration of 10 ng mL^−1^ at 37°C with 5% CO_2_ for 18 h. The cells were then washed with 1× PBS and incubated in glucose‐free RPMI 1640 medium (Gibco, US) containing 2‐NBDG (Cayman, Cambridge Bioscience, UK) at final concentration of 50 μm at 37°C with 5% CO_2_ for 30 min. The uptake of 2‐NBDG was measured by flow cytometry after surface staining.

For inhibition of memory‐like effector responses, unprimed or primed NK cells resuspended in R10 were plated at 250 000 cells per well in 96‐well round‐bottom plates in the presence and absence of metabolic inhibitors (described below) at 37°C with 5% CO_2_ for 18 h. Subsequently, PFA‐fixed bacteria and rhIL‐12 plus rhIL‐18 (as described above) were added to stimulate the cells at 37°C with 5% CO_2_ for 18 h. After stimulation, the cells were washed with 1× PBS and subjected to ECS and ICS for analysis of NK cell characteristics. The following inhibitors were added to the culture, and their final concentration was as follows: 2‐deoxy‐d‐glucose (2‐DG) (50 mm; Sigma), UK5099 (2 μm; Agilent Technologies, CA, US), bis‐2‐(5‐phenylacetamido‐1,3,4‐thiadiazol‐2‐yl) ethyl sulfide (BPTES) (3 μm; Agilent Technologies), Etomoxir (4 μm; Agilent Technologies) and Oligomycin A (1 nm; Sigma).

For analysis of memory‐like NK cell formation, the NK cell memory assay was performed, as described above. The following metabolic regulators were added to the culture from days 4 to 8, and their final concentration was as follows: 2‐DG (50 mm; Sigma), UK5099 (1 μm; Agilent Technologies, CA, US), BPTES (1.5 μm; Agilent Technologies), Etomoxir (2 μm; Agilent Technologies), Oligomycin A (0.5 nm; Sigma), metformin (1 μm; Merck Millipore, Darmstadt, Germany) and rapamycin (0.5 μm; Merck Millipore). Subsequently, cells were washed with 1× PBS and subjected to flow cytometry staining (ECS and ICS) and analysis.

### Detection of LC3‐II accumulation

LC3‐II accumulation was detected by using the Autophagy LC3 antibody‐based assay kit (Luminex Corporation, Austin, US). In brief, NK cells were treated with autophagy reagent A or lysosomal degradation inhibitor at 37°C with 5% CO_2_ for 4 h. Cells were washed with 1× PBS, subjected to ECS and washed with assay buffer in 96‐well round‐bottom plates. Autophagy reagent B (permeabilisation buffer) was added to each well and spun immediately, followed by staining with anti‐LC3‐FITC at 1:20 in assay buffer at 4°C for 30 min. Stained cells were washed with assay buffer and fixed with 2% paraformaldehyde (PFA) prior to flow cytometry analysis.

### Statistical analysis

All graphs and statistical analysis were performed using GraphPad Prism software version 9.0 (GraphPad Software Inc., La Jolla, CA, USA). Specifications of tests exploited and sample size for each experiment are mentioned in the respective figure caption. A non‐parametric Kruskal–Wallis test, followed by the Dunn's test with the Benjamini–Hochberg method for multiple comparisons, was employed to compare expression of nutrient transporters in healthy donors and melioidosis patients at different time points (day 0, 28‐day and 3‐month follow‐up). A non‐parametric Friedman's test, followed by the Dunn's test with Benjamini–Hochberg method for multiple comparisons, was performed to test for multiple dependent group differences between (1) melioidosis patients during acute disease and long‐term follow‐up and (2) primary NK cell experiments (unprimed and primed NK cells). A two‐tailed Wilcoxon matched‐pairs signed‐rank test was applied for comparing differences in CD160^−^ vs. CD160^+^ NK cells. Statistical significance was set at *P* < 0.05 and all tests were two‐tailed.

## Author contributions

**Anucha Preechanukul:** Conceptualization; formal analysis; funding acquisition; methodology; project administration; visualization; writing – original draft. **Natnaree Saiprom:** Formal analysis; methodology; writing – review and editing. **Kitilak Rochaikun:** Data curation; investigation; writing – review and editing. **Boonthanom Moonmueangsan:** Investigation. **Rungnapa Phunpang:** Data curation. **Orawan Ottiwet:** Investigation. **Yuphin Kongphrai:** Investigation. **Soonthon Wapee:** Investigation. **Rachan Janon:** Investigation; supervision; writing – review and editing. **Susanna Dunachie:** Investigation; resources; supervision; visualization; writing – review and editing. **Barbara Kronsteiner:** Conceptualization; formal analysis; methodology; supervision; visualization; writing – original draft. **Narisara Chantratita:** Conceptualization; funding acquisition; methodology; resources; supervision; visualization; writing – review and editing.

## Conflict of interest

The authors declare no conflict of interest.

## Supporting information


Supplementary figures 1–5
Supplementary tables 1–5

## Data Availability

All data are present in the article and its supplementary information files or from the corresponding author upon reasonable request. This paper does not report original code.
