# Supplementary material for: Metabolic requirements of CD160 expressing memory‐like NK cells in Gram‐negative bacterial infection
Source: Clin Transl Immunology. 2024 Jul 2;13(7):e1513. doi: 10.1002/cti2.1513 (PMC11218174; doi:10.1002/cti2.1513)
Supplement: Supplementary file 1 — Supplementary figures 1–5 Supplementary tables 1–5 [file CTI2-13-e1513-s001.docx]

**
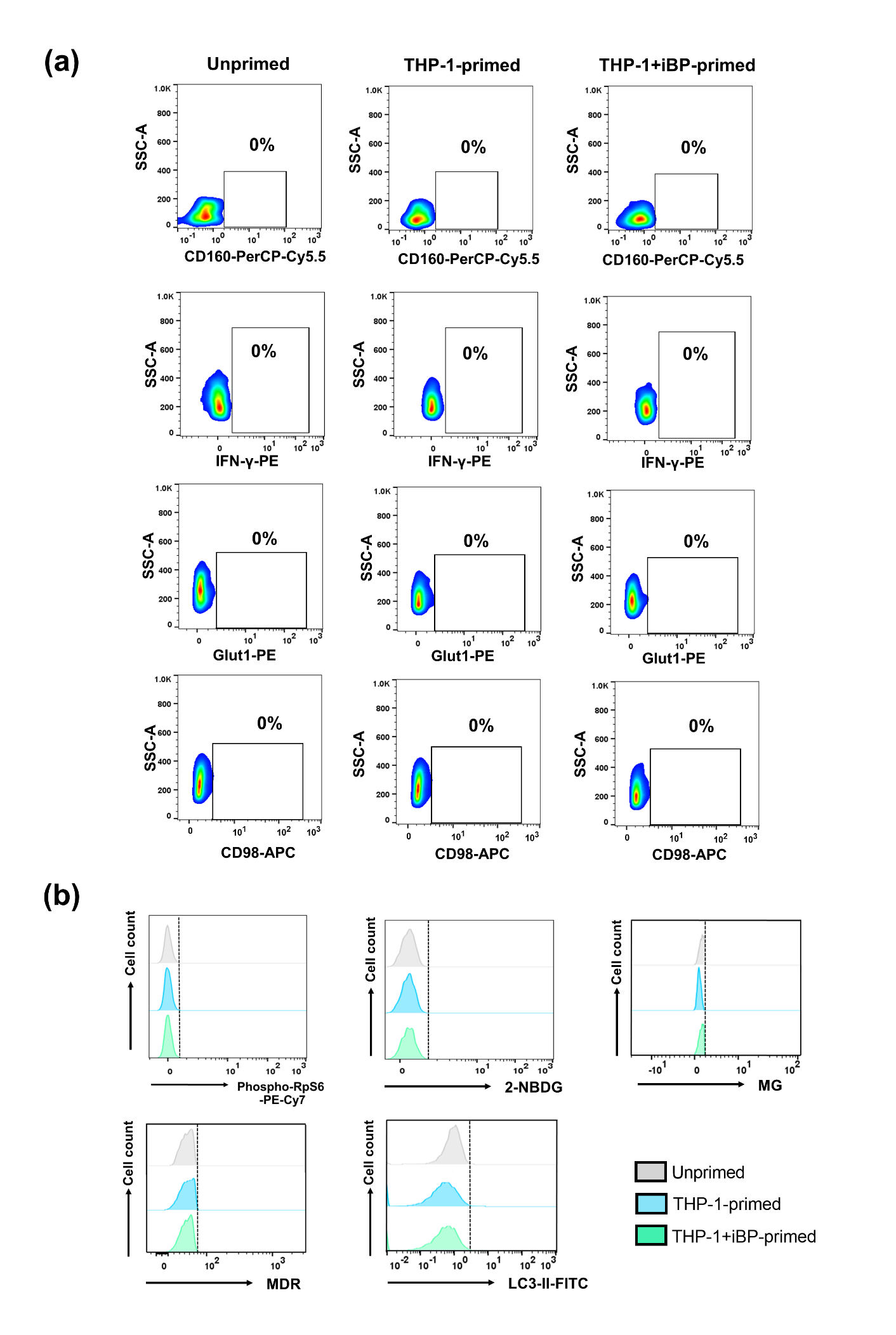
**

**Supplementary figure 1. Representative flow cytometry pseudocolor dot plots and histogram for metabolic characteristics of NK cells. (a)** Representative plots of fluorescence minus one (FMO) controls for CD160, IFN-γ, Glut1 and CD98 expression against SSC-A in unprimed and primed NK cells. **(b)** Representative histogram of FMO controls for Mitrotracker Green (MG), Mitotracker Deep Red (MDR), and LC3 in unprimed and primed NK cells.


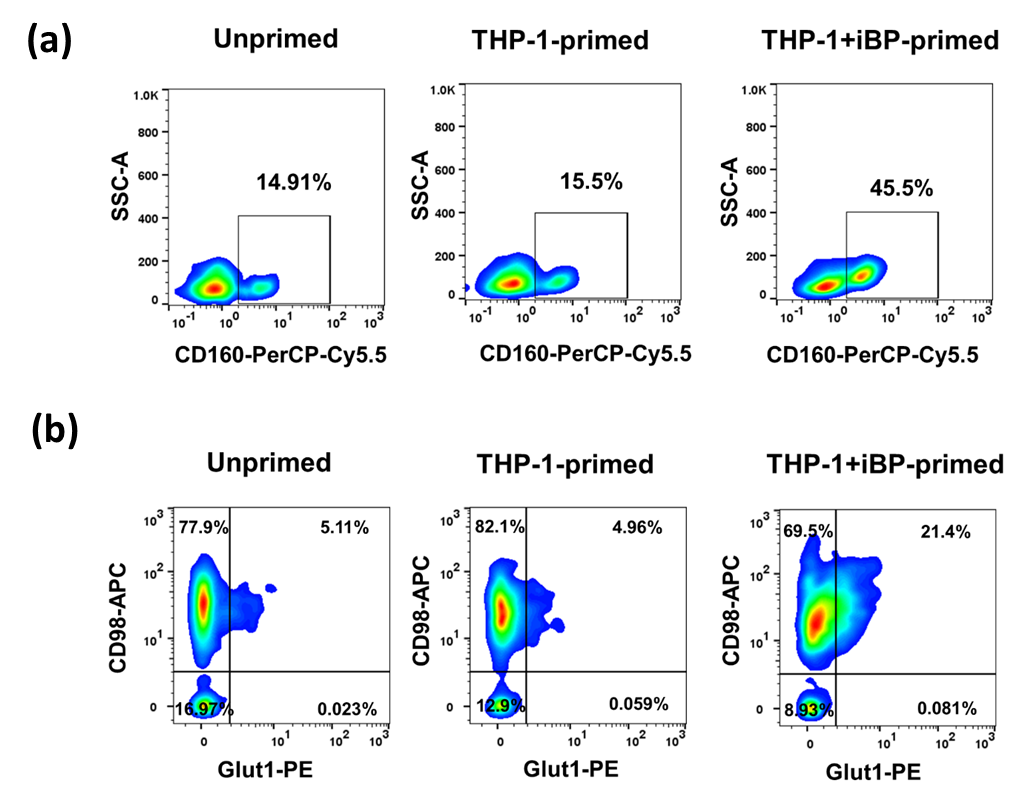


**Supplementary figure 2. Expression of CD160, amino acid and glucose transporters on memory-like NK cells.** Representative flow cytometry dot plots for **(a)** CD160 expression **(b)** CD98 and Glut1 co-expression in unprimed and primed NK cells.


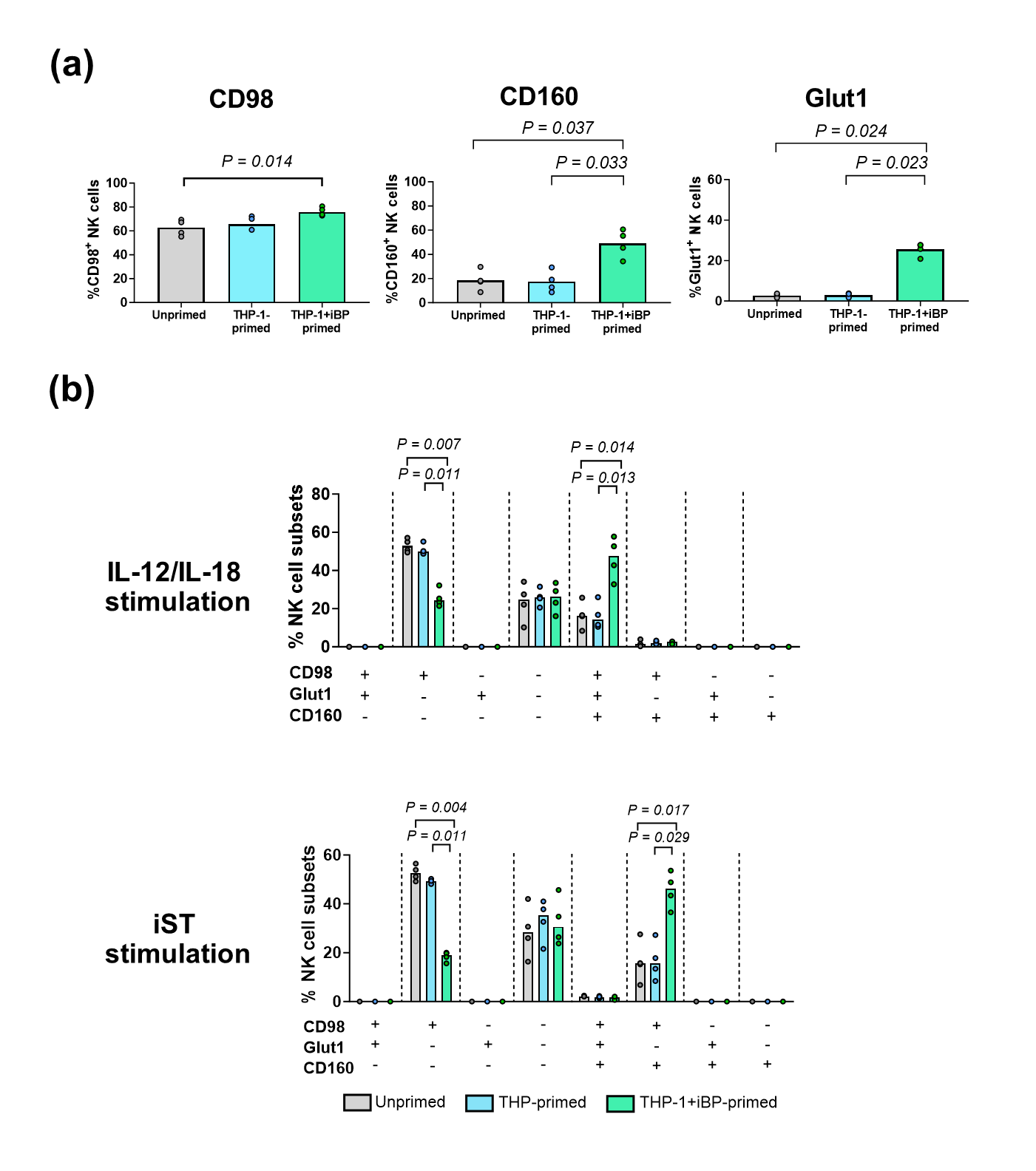


**Supplementary figure 3. Expression of CD160, amino acid and glucose transporters on NK cells upon stimulation.** **(a)** The percentage of unprimed-, THP-1-primed- or THP-1+iBP-primed NK cells expressing CD98, CD160 and Glut1 in response to iBP stimulation. **(b)** The proportion of CD160^-^ and CD160^+^ NK cells co-expressing CD98 and Glut1 upon IL-12/IL-18 or inactivated *Salmonella enterica* serovar Typhi (iST) stimulation. Data from 4 individual donors are presented in bar graphs. Each data point represents the median of three technical replicates (independent experiments) for each healthy donor. The medians of the technical replicates were used for statistical testing and graphical presentation. Statistical analysis was performed using the Friedman’s test, followed by the Dunn’s test with the Benjamini-Hochberg method for multiple comparison, and only *P*-values for statistically significant (*P < 0.05*) comparisons are shown on the graphs.


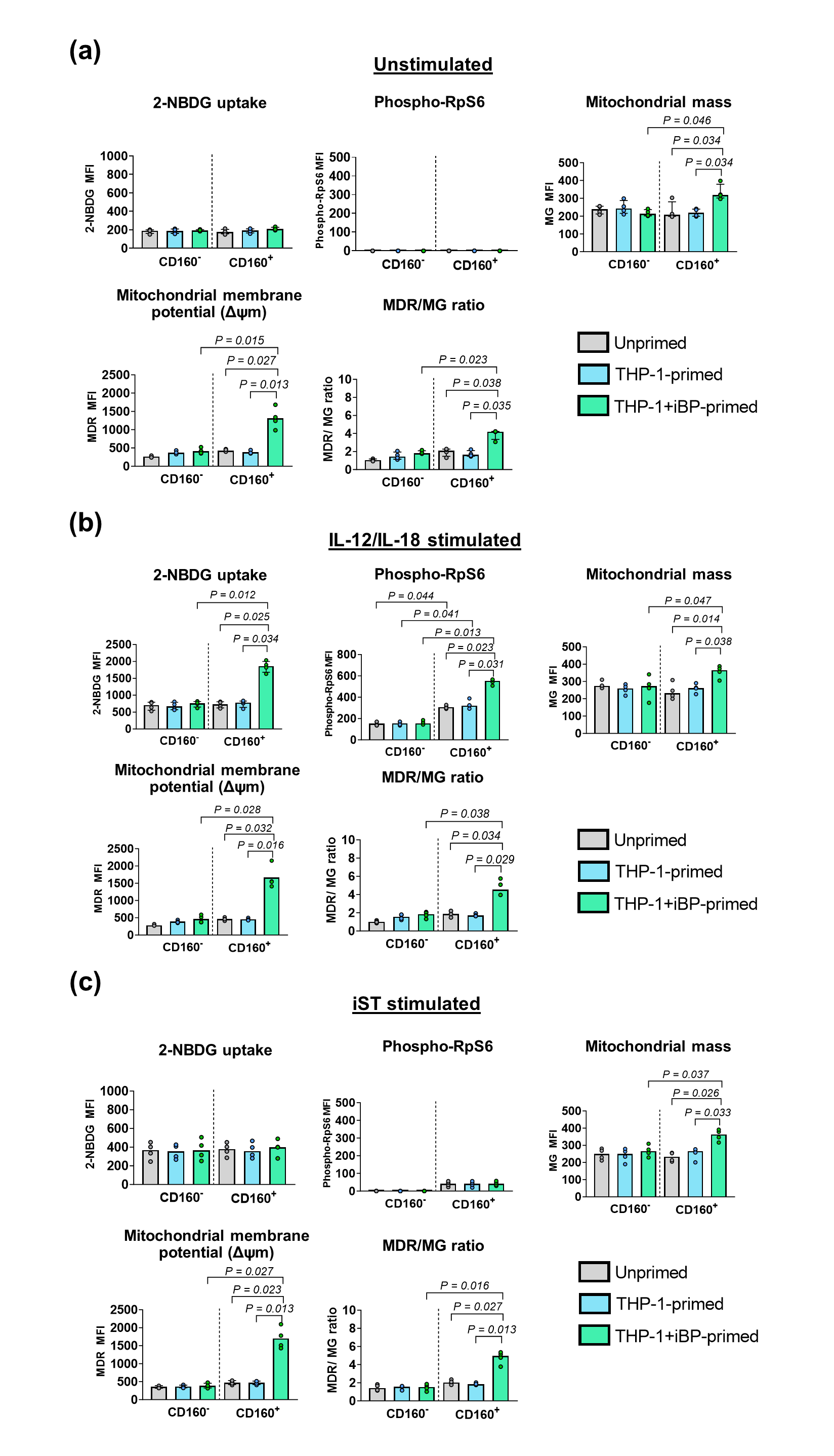


**Supplementary figure 4. Metabolic characteristics of memory-like NK cells upon IL-12/IL-18 and iST stimulation.** Unprimed-, THP-1-primed or THP-1+iBP-primed NK cells were stimulated with IL-12/IL-18 or inactivated *Salmonella enterica* serovar Typhi (iST) iST for 18 h. Metabolic features of NK cells were determined by flow cytometry. Metabolic features of unprimed and primed NK cells in CD160^-^ and CD160^+^ subsets: Median fluorescence intensity (MFI) of 2-NBDG (uptake of glucose analogue), phosphorylated RpS6, mitochondrial mass, and mitochondrial membrane potential (Δψm), and MitoTracker Deep Red (MDR) normalized by MitoTracker Green in **(a)** unstimulated-, **(b)** IL-12/IL-18 stimulated- or **(c)** iST stimulated conditions. Data from 4 individual donors are presented in bar graphs. Each data point represents the median of three technical replicates (independent experiments) for one healthy donor. The medians of the technical replicates were used for statistical testing and graphical presentation. Statistical analysis was performed using the Friedman’s test, followed by the Dunn’s test with the Benjamini-Hochberg method for multiple comparison, and only *P*-values for statistically significant (*P <* 0.05) comparisons are shown on the graphs.

**
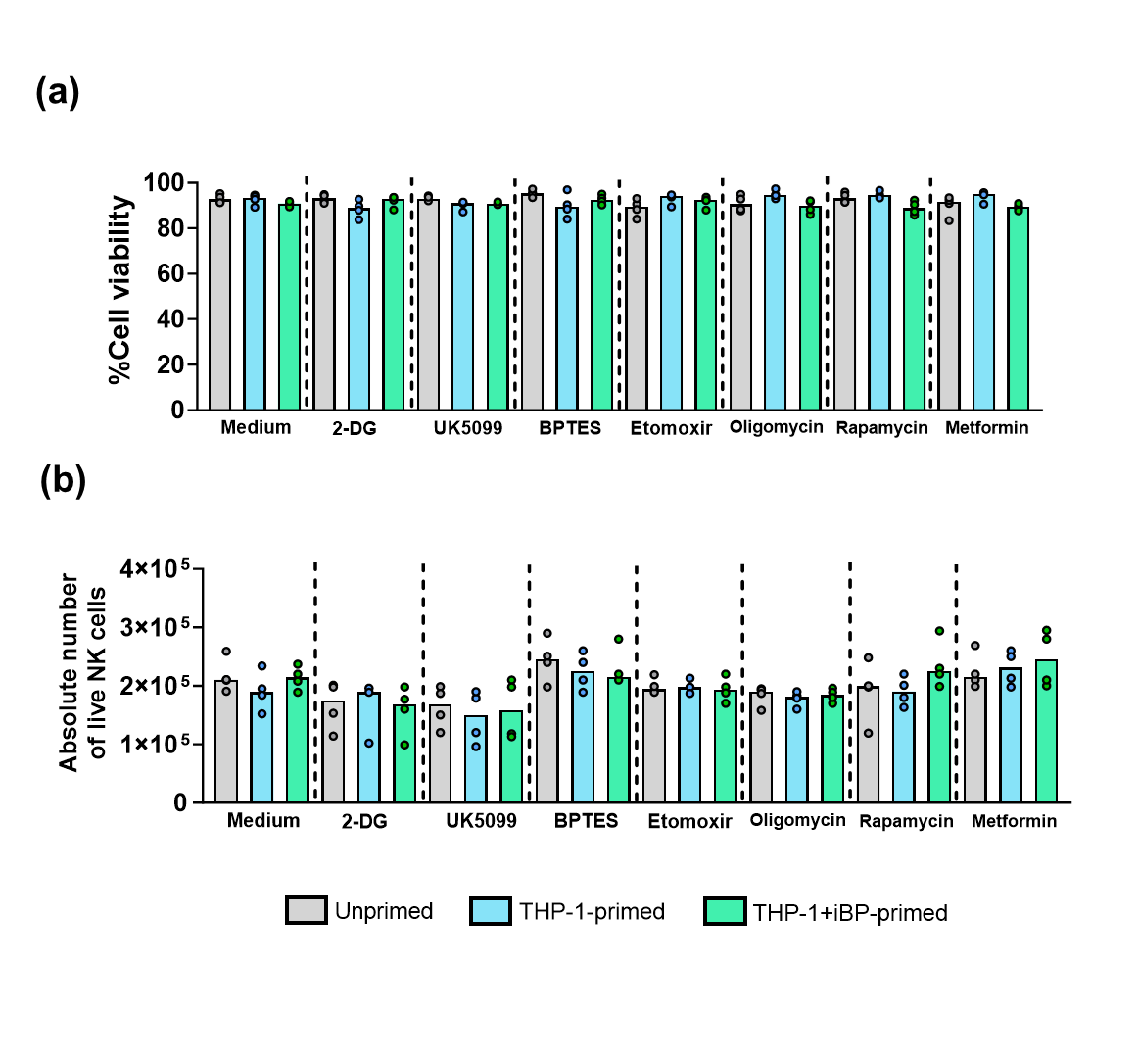
**

**Supplementary figure 5.** **Metabolic inhibitors did not affect the NK cell viability and the absolute number of live NK cells.** The NK cell memory assay was performed to generate memory-like NK cells. **(a)** The percentages of NK cell viability and **(b)** the absolute number of live NK cells in the absence or presence of metabolic regulators at 4 days post priming are shown in unprimed and primed conditions. Data from 4 individual donors are presented in bar graphs. Each data point represents the median of three technical replicates (independent experiments) for each healthy donor. The medians of the technical replicates were used for statistical testing and graphical presentation. Statistical analysis was performed using the Friedman’s test, followed by the Dunn’s method with the Benjamini-Hochberg method for multiple comparison, and only *P*-values for statistically significant (*P <* 0.05) comparisons are shown on the graphs.

**Supplementary table 1**. Demographic characteristics of melioidosis and healthy donors in a Thai cohort

| **Characteristics** | **Melioidosis patients**  **(n = 20)** | **Healthy Donors (n = 30)** |
| --- | --- | --- |
| Age, median (IQR) | 51(38–56) | 42(37–48) |
| Sex Male/Female(M%) | 15/5 (75) | 12/18 (40) |
| Bacteremia (%) | 14 (70) | N/A |
| Cardiovascular disease (%) | N/A | N/A |
| Pre-existing liver disease (%) | 2(10) | N/A |
| Pre-existing renal disease (%) | 1 (5) | N/A |
| Diabetes (%) | 14 (70) | N/A |
| Hypertension (%) | 5(25) | N/A |
| Pneumonia (%) | 4(20) | N/A |
| Previous history of melioidosis | N/A | N/A |
|  |  |  |

**Supplementary table 2**. The percentage of IFN-γ^+^ CD160^+^ subset within a total population of unprimed, THP-1 primed and THP-1+iBP primed NK cells upon IL-12/IL-18 stimulation in absence and presence of metabolic inhibitors.

| **NK cells** | **Metabolic inhibitors** | | | | | |
| --- | --- | --- | --- | --- | --- | --- |
|  | **Medium** | **2DG** | **UK5099** | **BPTES** | **Etomoxir** | **Oligomycin** |
| Unprimed | 18.83 | 11.97 | 20.57 | 18.37 | 19.70 | 19.53 |
|  | 22.00 | 9.35 | 24.07 | 20.05 | 22.07 | 17.80 |
|  | 23.10 | 14.30 | 21.48 | 18.97 | 20.57 | 18.87 |
|  | 25.47 | 13.03 | 26.99 | 15.73 | 24.50 | 20.57 |
|  |  |  |  |  |  |  |
| THP-1 primed | 20.47 | 15.00 | 20.21 | 17.59 | 20.07 | 19.53 |
|  | 20.73 | 10.42 | 20.29 | 17.54 | 19.63 | 17.80 |
|  | 19.99 | 11.47 | 19.40 | 18.16 | 19.76 | 18.87 |
|  | 22.93 | 11.77 | 21.59 | 21.39 | 26.63 | 20.57 |
|  |  |  |  |  |  |  |
| THP-1+iBP primed | 41.35 | 30.07 | 39.97 | 34.58 | 41.50 | 19.32 |
|  | 47.22 | 34.87 | 42.40 | 36.50 | 41.70 | 14.57 |
|  | 39.33 | 38.83 | 43.27 | 36.14 | 39.16 | 14.42 |
|  | 39.17 | 31.07 | 39.03 | 37.47 | 41.23 | 13.01 |
|  |  |  |  |  |  |  |

**Supplementary table 3**. The percentage of IFN-γ^+^ CD160^-^ subset within a total population of unprimed, THP-1 primed and THP-1+iBP primed NK cells upon IL-12/IL-18 stimulation in absence and presence of metabolic inhibitors.

| **NK cells** | **Metabolic inhibitors** | | | | | |
| --- | --- | --- | --- | --- | --- | --- |
|  | **Medium** | **2DG** | **UK5099** | **BPTES** | **Etomoxir** | **Oligomycin** |
| Unprimed | 14.80 | 7.93 | 16.53 | 14.33 | 15.67 | 15.43 |
|  | 16.32 | 3.67 | 18.39 | 14.37 | 16.39 | 14.42 |
|  | 17.75 | 8.95 | 16.13 | 13.61 | 15.21 | 15.96 |
|  | 17.48 | 5.05 | 19.00 | 11.75 | 16.51 | 16.71 |
|  |  |  |  |  |  |  |
| THP-1 primed | 16.43 | 9.97 | 16.18 | 13.56 | 16.03 | 15.50 |
|  | 15.05 | 9.41 | 14.61 | 11.86 | 13.95 | 12.12 |
|  | 14.64 | 7.37 | 14.05 | 12.80 | 14.41 | 13.51 |
|  | 14.95 | 9.45 | 13.60 | 13.41 | 18.65 | 12.58 |
|  |  |  |  |  |  |  |
| THP-1+iBP primed | 17.32 | 11.03 | 15.93 | 13.22 | 17.47 | 15.29 |
|  | 15.54 | 12.19 | 16.72 | 14.15 | 16.02 | 10.23 |
|  | 17.31 | 7.48 | 17.91 | 14.12 | 13.80 | 11.07 |
|  | 17.51 | 11.97 | 14.38 | 15.15 | 13.25 | 14.69 |
|  |  |  |  |  |  |  |

**Supplementary table 4**. The percentage of IFN-γ expression in the CD160^+^ and CD160^-^ subsets of THP-1+iBP primed NK cells upon iBP stimulation in absence and presence of metabolic inhibitors. iBP = inactivated *B. pseudomallei*

| **THP-1+iBP primed NK cells** | **Metabolic inhibitors** | | | | | |
| --- | --- | --- | --- | --- | --- | --- |
|  | **Medium** | **2DG** | **UK5099** | **BPTES** | **Etomoxir** | **Oligomycin** |
| IFN-γ^+^CD160^+^ | 19.70 | 13.03 | 20.93 | 14.80 | 20.81 | 1.07 |
|  | 15.43 | 10.67 | 18.60 | 12.53 | 17.30 | 1.83 |
|  | 11.70 | 9.20 | 16.10 | 12.47 | 13.77 | 2.30 |
|  | 19.53 | 11.60 | 18.63 | 12.23 | 20.20 | 0.47 |
| IFN-γ^+^CD160- | 0.60 | 0.45 | 0.60 | 0.47 | 0.48 | 0.50 |
|  | 0.43 | 0.33 | 0.48 | 0.30 | 0.47 | 0.47 |
|  | 0.70 | 0.57 | 0.56 | 0.77 | 0.77 | 0.53 |
|  | 0.60 | 0.60 | 0.53 | 0.57 | 0.57 | 0.50 |

**Supplementary table 5.** Reagents and fluorochrome-conjugated monoclonal antibodies (mAbs) for the analysis of NK cell phenotype and function

| **Markers** | **Fluorochrome**  **/Reporter** | **Clone** | **Target species** | **Host species** | **Isotype** | **Manufacturer** | **Stock conc.**  **(µg mL^-1^)** | **Final**  **conc.**  **(µg mL^-1^)** | **Optimal dilution** | **Characteristic measured** |
| --- | --- | --- | --- | --- | --- | --- | --- | --- | --- | --- |
| CD3 | BV711 | OKT3 | Human | Mouse | IgG1, κ | Biolegend | 40 | 0.4 | 1:100 | Cell surface protein |
| CD3 | APC-Fire750 | 3G8 | Human | Mouse | IgG1, κ | Biolegend | 200 | 2 | 1:100 | Cell surface protein |
| CD14 | APC-Fire750 | M5E2 | Human | Mouse | IgG1, κ | Biolegend | 400 | 2 | 1:200 | Cell surface protein |
| CD19 | APC-Fire750 | HIB19 | Human | Mouse | IgG1, κ | BIolegend | 200 | 1.33 | 1:150 | Cell surface protein |
| CD36 | PE-Cy7 | 5-271 | Human | Mouse | IgG1, κ | BIolegend | 12 | 0.3 | 1:40 | Cell surface protein |
| CD56 | BV605 | HCD56 | Human | Mouse | IgG1, κ | Biolegend | 100 | 3.03 | 1:33 | Cell surface protein |
| CD56 | BV421 | HCD56 | Human | Mouse | IgG1, κ | Biolegend | 60 | 1.2 | 1:50 | Cell surface protein |
| CD98 | APC | REA387 | Human | Human | IgG | Miltenyibiotec | NA | NA | 1:50 | Cell surface protein |
| CD98 | FITC | MEM-108 | Human | Mouse | IgG1, κ | Biolegend | 200 | 5 | 1:40 | Cell surface protein |
| CD160 | PerCP-Cy5.5 | 7H1 | Human | Mouse | IgG1, κ | Biolegend | 200 | 6.67 | 1:30 | Cell surface protein |
| CD160 | AF488 | BY155 | Human | Mouse | IgG1, κ | BD Bioscience | 100 | 3.33 | 1:30 | Cell surface protein |
| Glut1 | PE | EPR3915 | Human | Rabbit | IgG | Abcam | 500 | 25 | 1:20 | Cell surface protein |
| IFN-γ | PE | 4S.B3 | Human | Mouse | IgG1, κ | Biolegend | 10 | 0.25 | 1:40 | Intracellular protein |
| RPS6 Phospho | PE-Cy7 | A17020B | Human | Mouse | IgG1, κ | Biolegend | 6 | 0.3 | 1:20 | Intracellular protein  Phospho-protein |
| IFN-γ | PE | 4S.B3 | Human | Mouse | IgG1, κ | Biolegend | 10 | 0.25 | 1:40 | Intracellular protein |
| Fixable Near IR Dead Cell | Near IR | NA | Human | Mouse | NA | Invitrogen | NA | NA | 1:1000 | Cell death |
